# Inhibiting the Ins and Outs of HIV Replication: Cell-Intrinsic Antiretroviral Restrictions at the Plasma Membrane

**DOI:** 10.3389/fimmu.2017.01853

**Published:** 2018-01-04

**Authors:** Toshana L. Foster, Suzanne Pickering, Stuart J. D. Neil

**Affiliations:** ^1^Department of Infectious Disease, School of Immunology and Microbial Sciences, King’s College London, London, United Kingdom

**Keywords:** human immunodeficiency virus, type I interferons, antiviral restriction, plasma membrane, tetherin/BST-2, serine incorporator, interferon-induced transmembrane

## Abstract

Like all viruses, human immunodeficiency viruses (HIVs) and their primate lentivirus relatives must enter cells in order to replicate and, once produced, new virions need to exit to spread to new targets. These processes require the virus to cross the plasma membrane of the cell twice: once via fusion mediated by the envelope glycoprotein to deliver the viral core into the cytosol; and secondly by ESCRT-mediated scission of budding virions during release. This physical barrier thus presents a perfect location for host antiviral restrictions that target enveloped viruses in general. In this review we will examine the current understanding of innate host antiviral defences that inhibit these essential replicative steps of primate lentiviruses associated with the plasma membrane, the mechanism by which these viruses have adapted to evade such defences, and the role that this virus/host battleground plays in the transmission and pathogenesis of HIV/AIDS.

## Introduction

A key feature of eukaryotic cells is the plasma membrane (PM), the single lipid bilayer that delimits the cytoplasm from the extracellular milieu (1). As well as acting as the physical boundary of the cell, the PM acts as a platform which plays a role in almost every cellular process, from regulating transport of small molecules and proteins in out of the cell, to cell mobility, and the response to its environment. As such, any infectious agent that seeks to gain access to the cell’s cytosol must breach the PM or the limiting membranes of intracellular compartments. In the case of enveloped viruses, this entails an entry step in which viral envelope glycoproteins engage specific cellular receptors on the PM or undergo low pH-induced conformational changes upon endocytic uptake (2, 3). As a result of either of these processes, mechanisms intrinsic to the glycoproteins themselves mediate fusion between the viral and host cell membranes, allowing the viral genetic material to enter the cell and initiate the replication cycle. For lentiviruses, the replication cycle culminates in newly synthesized RNA genomes and viral structural proteins being targeted to the inner leaflet of the PM (4). With the aid of a multitude of cellular factors, new virus particles assemble and bud into the extracellular space, acquiring their lipid envelope from the host cell. Budding ends in a scission event that separates the new virion from the cell, allowing it to be released and infect new targets.

These unavoidable processes are common to all enveloped viruses. Moreover, the lipid envelope is the one component of the virus particle that is not encoded by the virus itself. It is perhaps unsurprising that the mammalian host has evolved multiple antiviral mechanisms whose role is to inhibit viral replicative processes that are associated with entry and exit (5–8), necessitating either the evolution of directly encoded countermeasures by the virus, or other mechanisms of resistance or avoidance. Furthermore, these mechanisms are often (but not always) regulated by type 1 interferons (IFN-I) and pattern recognition responses, linking these factors to the wider antiviral immune response.

## Overview of Lentiviral Entry and Exit

The mediator of the entry of HIV-1 and its related viruses is the trimeric envelope spike [reviewed in Ref. (9)]. For HIV-1, this is comprised of three precursor Env proteins, gp160, that are proteolytically cleaved into a surface subunit, gp120, and a transmembrane subunit gp41. gp120 harbours the receptor binding components of the envelope spike whereas gp41 encodes the fusion machinery itself, buried within the trimer. gp120 consists of a series of conserved domains interspersed with variable loops and is heavily glycosylated on the outer faces of the trimer (10). There are surprisingly few spikes on the surface of the virion, with estimates of about 10–20 (11). Super-resolution microscopy imaging of HIV-1 particles has shown that these spikes cluster, which appears to be important for fusogenicity (12).

The Env trimer is a metastable structure, poised to mediate viral entry upon interaction with its receptor(s) (9). When gp120 binds to its cognate receptor, CD4 (13–15), on the target T cell or macrophage, structural rearrangements “open” the envelope to reveal a coreceptor binding site (16–18). This interacts with either CCR5 (19–21) or CXCR4 (22), and occasionally additional CC chemokine receptors. Upon coreceptor binding, further conformational changes expose the hydrophobic fusion peptide of gp41, which rapidly inserts in the target membrane. The extended conformation of the gp41 trimer collapses back to form a six-helix bundle common to diverse type 1 enveloped virus fusion proteins (9). This pulls the viral and cellular membranes together, and is sufficient to locally destabilise the membranes, allowing lipid mixing, fusion, and the release of the viral core into the cell (9).

The use of CCR5 appears to be essential for sexual transmission of HIV-1. Viruses that use CCR5 alone (R5), or more rarely CCR5 and CXCR4 [R5/X4 or dual tropic (23)], predominate in early infection (24, 25). Individuals homozygous for a 32 base pair deletion in CCR5 that disrupts its expression are largely HIV-1 resistant (26, 27). X4-using viruses tend to arise later in infection in some, but not all, individuals, and are associated with more rapid progression to AIDS. Whilst they can be transmitted by intravenous drug-use/transfusion, it is not clear why X4 viruses are almost never transmitted sexually given that target CD4+ T cells in the mucosa express CXCR4 (25). The selective pressures that produce the so-called coreceptor switch are not well understood, but it is associated with changes in the V3 loop of gp120 and perhaps arises through escape from certain classes of neutralizing antibody (28). Coreceptor usage in part determines the cellular tropism of the virus; R5 viruses infect predominantly subsets of antigen-experienced CD4+ T cells, whereas X4 usage expands this tropism to naïve cells (9). Macrophage-tropic viruses are almost exclusively R5 users, but importantly macrophage tropism is determined by changes in gp120 that allow it to use much lower cell surface concentrations of CD4 (29, 30). Thus most R5 isolates, including those transmitted between individuals (the so called transmitted-founder (TF) viruses) can only infect T cells (31). Quite why the majority of X4 viruses cannot infect macrophages which express abundant CXCR4 is not known (32).

Where entry occurs in the cell has been of some controversy. The pH-independence of HIV-1 entry would suggest that it occurs at the cell surface (2, 33). This was reinforced by early studies showing that endocytosis of CD4 was not necessary for productive viral entry (34). However, more recent studies have shown that HIV-1 entry is sensitive to certain endocytosis inhibitors, particularly those targeting the GTPase dynamin-2 (35). These effects may be cell-type dependent, as entry appears to be predominantly cell surface in T cell lines (36). Furthermore dynamin-2 may play a role in fusion, independent of its activity in endocytosis (37). Much further work, particularly with clinically relevant isolates, is required to fully rationalize many of these observations. However, the ability of certain membrane associated antiviral factors to differentially restrict HIV-1 entry dependent on their own subcellular localization may allow further insight into these issues.

The next encounter of HIV-1 with the limiting membrane of the cell is viral assembly [reviewed in Ref. (4, 38)]. For lentiviruses, this occurs exclusively at the plasma membrane. Small amounts of Gag and Gag-Pol polyproteins are targeted to the inner leaflet of the PM, bringing with them two copies of the viral genomic RNA. This allows more Gag/Gag-Pol to nucleate around them, and in doing so form a budding virion. Small peptide motifs in the p6 portion of Gag (termed late domains) interact with several members of the ESCRT pathway, a multi component protein machinery that resolves membrane-bound entities budding away from the cell’s cytoplasm. The recruitment of the core ESCRT-I subunit TSG101 is the major event in initiating HIV-1 release, although other associated factors can also directly interact with Gag. This then leads to the recruitment of charged multivesicular protein (CHMP) subunits of ESCRT-III. The polymerization of these ESCRT-III subunits into filaments around the inside of the stem of the budding virions and their subsequent depolymerization by the AAA-ATPase VPS4, leads to the contraction of the neck of the bud and the final scission of the virus from the cell. During the budding process, mature Env trimers are recruited into the assembling virion, as well as a number of other host membrane proteins; some beneficial, others, as described below, less so. Co-incident with the latter stages of budding, dimerization of the protease component of the Gag-Pol polyprotein, driven by interactions between reverse transcriptase moieties, activates its catalytic activity. This then leads to the sequential processing of the Gag and Gag-Pol to generate the mature structural and enzymatic components of the infectious virion.

## Type 1 Interferons and the Restriction of HIV-1 Replication

A burst of systemic inflammatory cytokines driven by type 1 interferons (IFN-I) is one of the earliest host responses detectable in HIV-1 infected individuals (39). Despite the virus being adept at avoiding host pattern recognition receptors in infected cells (see review by Sumner et al. in this issue), the consequence of the rapid increase in viral replication is that systemic IFN-I levels are detectable as early as 7 days after infection. Both alpha and beta interferons activate the same receptor, IFNAR1/2, expressed on the majority of somatic cells, and via the Jak/STAT pathway induce the transcription of hundreds of so-called interferon-stimulated genes (ISGs), many of which, like IFN-I themselves, are also activated directly by pattern recognition responses (40). In addition to the activation of systemic innate and adaptive immunity, a number of these ISGs have direct antiviral activity against the replicative stages of diverse mammalian viruses (7). These antiviral factors, sometimes called restriction factors, often target common pathways or structures that are essential for viral replication, and which cannot be simply mutated around. In the case of lentiviruses, several restriction factors have been identified that are targets of virally encoded accessory proteins (41), for example tetherin and Vpu described below. The evolutionary arms race between these countermeasures and species-specific orthologues of these restriction factors has shaped the adaptation of these viruses to new primate hosts, ultimately allowing chimpanzee and sooty mangabey simian immunodeficiency viruses to cross into humans to become HIV-1 and HIV-2 respectively (42). However, ectopic expression of a number of ISGs have a direct antiviral activity against HIV-1 with no obvious virally-encoded countermeasure (43). HIV-1 replication can be inhibited in primary CD4+ T cells and macrophages in culture by IFN-I treatment, indicating some of these ISGs may play a physiological role in early infection (43, 44). Furthermore, treating HIV-infected patients with pegylated-IFN leads to a transient reduction in viral loads (45). In macaques, although initial mucosal inflammasome activation may inhibit local ISG activation (46), early viremic control of SIVmac infection is dependent on systemic IFN-I responses (47). But perhaps the most powerful evidence of the importance of directly antiviral ISGs in HIV-1 pathogenesis comes from the observation that viruses that represent the most likely founder of an individual’s infection, called transmitted/founder (TF) viruses, display a considerably higher resistance to the effects of IFN-I in their replication in primary CD4+ T cells than viruses isolated during the chronic phase (31, 48). While initially controversial in a replication study in subtype C infections using blood-derived viral sequences (49), these observations have been extended and now show that the TF virus sequence in a recipient partner is the most IFN-I resistant amongst the viral quasi-species that existed in the donor partners’ genital secretions at the time of transmission in both clades B and C, thus indicating IFN-I resistance is a key attribute for transmission fitness (50). Curiously, as infection progresses, IFN-I resistance in circulating virus wanes (48). There are multiple molecular determinants of this difference in IFN sensitivity between TF and chronic viruses from the same the donor, suggesting a number of ISGs are involved (50). In the sections below, we will discuss host restriction factors and antiviral ISGs that target the entry and exit pathways of the virus.

## The Interferon-Induced Transmembrane (IFITM) Protein Family

The interferon-induced transmembrane (IFITM) proteins are a family of antiviral factors that restrict the fusion of a number of pathogenic enveloped viruses with their target cells, including influenza A virus (IAV), Dengue virus (DENV), hepatitis C virus (HCV), Ebola virus (EBOV) and HIV (51–53). They are predominantly located at the PM and on endosomal membranes, the portals of entry for most viruses (54, 55). Recent studies have sought to identify the mechanisms of their antiviral restriction activities that may explain this broad spectrum activity, which primarily target the entry stages of the viral lifecycle.

Five members of the gene family have been identified in humans, *ifitm 1, 2, 3, 5 and 10*, all clustered on chromosome 11 (56, 57). Unlike *ifitms 1, 2* and *3, ifitm5* is not induced by type 1 or type 2 interferons but has been proposed to be involved in bone mineralization. A function for *ifitm10* has not been identified. In the mouse genome, the orthologues of the human *ifitm* genes are located on chromosome 7, with the pseudogene *ifitm4*, also not functional in humans, located in close proximity to *ifitm 1, 2 and 5*. Analogous genes have been identified in other mammals and in the avian species, where the IFITM proteins serve to inhibit influenza viruses.

### IFITM Structure and Localisation

**Figure 1:**
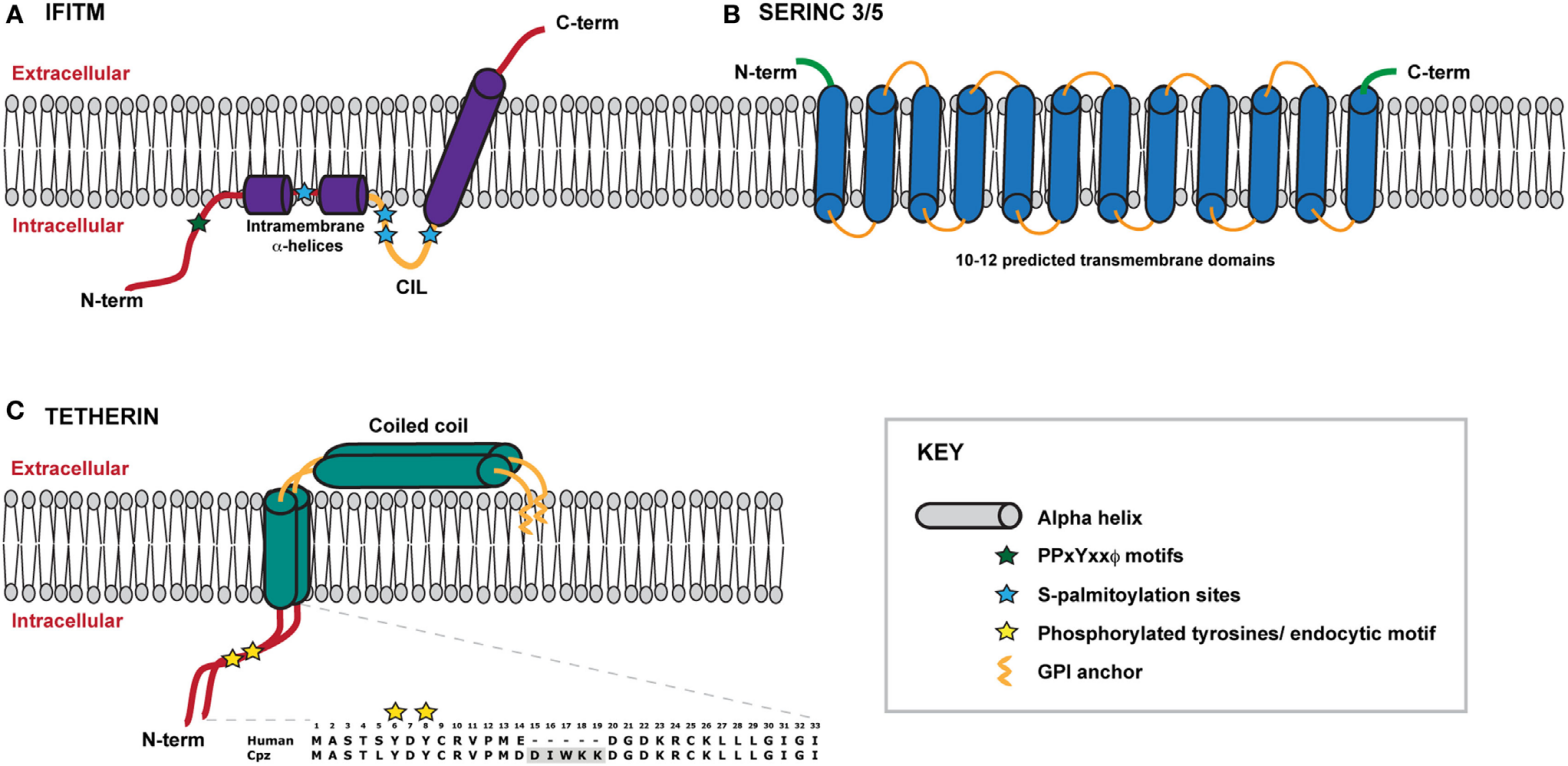
Schematic representation of IFITMS, SERINC 3/5 and tetherin within a model membrane. **(A)** A model of the IFITM protein, which adopts a type II transmembrane protein topology in the membrane. The N-terminal domain lies within the cytoplasm and connects to two short intramembrane α-helices. IFITMs 2 and 3 possess a longer N-terminal domain that contains important trafficking motifs that determine protein localisation. The conserved intracellular loop (CIL) contains sites of palmitoylation that likely stabilise the conformation of the C-terminal transmembrane α-helix which spans the membrane, thus positioning the C-terminal domain within the extracellular space. **(B)** Very little information about the structures of SERINC proteins is currently known. SERINCs 3 and 5 are thought to possess between 10 and 12 transmembrane helices such that the N- and C-termini reside within the extracellular space. **(C)** Tetherin exists as a dimer anchored to the membrane via an N-terminal transmembrane domain and a C-terminal GPI anchor. The extracellular portion of tetherin is comprised of a coiled coil. The N terminal cytoplasmic tail contains a dual tyrosine motif that plays a role in both steady-state cycling of the protein and signal transduction following virus retention. Amino acid sequences of human and chimpanzee cytoplasmic tails are shown for comparison, highlighting the deletion of the DIWKK motif. The short isoform of human tetherin lacks the first 12 amino acids of the cytoplasmic tail.

IFITMs are members of a larger superfamily of proteins found in both eukaryotes and prokaryotes, known collectively as dispanins (58). Structurally, the IFITMs each contain two hydrophobic domains that are separated by a short conserved intracellular loop (CIL) containing a CD225-like domain; speculation has however surrounded the topological conformation of the domains within the membrane. The current biochemical and cell biology evidence suggest that the IFITMs adopt a topology in which the N-terminus and CIL reside in the cytoplasm, with the first hydrophobic domain existing as an intra-membrane domain whilst the second hydrophobic domain spans the membrane such that the C-terminus resides in the extracellular space (Figure 1A) (54, 55, 59). The CIL domain also contains palmitoylation sites that likely stabilize this conformation (60–62). The intramembrane helices of the first hydrophobic domain are postulated to influence the curvature of the membrane in which the IFITM resides thus impacting the restriction activity (59). Evidence of self-association and intramolecular interactions between the IFITM proteins, via residues within the first transmembrane domain, has been reported, suggesting that higher order multimers may have functional implications (63).

Mammalian IFITMs are highly homologous at the amino acid level, and in particular IFITMs 2 and 3 in primates display highly complex positive selection signatures (64) suggesting that they are continually adapting to target pathogenic viruses (65). Such selection raises the notion that they may be under pressure to provide a continuous barrier across the entry portals into the cell. Consistent with this, human IFITMs localize to distinct but overlapping cellular membranes (54). While IFITM1 appears to be mainly associated with the PM, the longer N-terminal cytoplasmic tail of IFITMs 2 and 3 contain a YxxΦ endocytic motif that permits their localization to early/recycling (IFITM3) and late (IFITM2) endosomal compartments (66, 67). This sorting signal overlaps with an endosomal degradation motif (PPxY) that regulates their turnover (Figure 1A) (68). Importantly, therefore, both endosomal IFITMs dynamically traffic via the cell surface to reach their major sites of localization. This localization is a key determinant of the antiviral spectrum which a given IFITM restricts because the mechanism of entry of different viruses (receptor requirements, pH thresholds of fusion etc.) define their sites of access to the cell. For example, mutation of the endocytic motif in IFITM3 such that it redistributes to the cell surface abolishes its antiviral activity against IAV (67). This has major implications for the discussion of their effects on HIV below.

### Mechanism of IFITM Restriction

IFITMs appear to block the physical fusion of enveloped viruses with their target membranes, however the mechanism of action is not clear. It is widely postulated from the work of Brass, Liu and others particularly on IAV, that the mechanism of action is through modulation of the host cell membrane fluidity to block viral fusion (69–73). These “tough-membrane” models suggest a number of possible mechanisms: (1) Adjacent IFITM molecules may interact via their intramembrane domains thereby decreasing the fluidity of the host membrane and limiting the lateral movement of host entry receptors and formation of productive receptor complexes. (2) These intramolecular interactions may prevent the effective viral envelope clustering that is required particularly for IAV fusion and (3) the IFITM multiplexes could also form a “meshwork” within the outer leaflet of the membrane that not only decreases fluidity and imposes rigidity but induces an outward membrane curvature that opposes the forces exerted by the viral fusion machinery. These general mechanisms may account for the diversity of viruses inhibited, including non-enveloped viruses, such as reoviruses, that do not require fusion, but do need to disrupt the endosomal membrane to enter the cell (74). Such models are also consistent with observations of IAV and Semliki Forest virus (SFV) accumulating in endosomal compartments where the restricting IFITM resides, without affecting the pH-dependent exposure of the viral fusion machinery (70, 71, 75). Studies have demonstrated that IFITM-mediated restrictions of fusion can be overcome by antifungal drugs that target cholesterol metabolism, and oelic acid treatment that is predicted to reverse the positive membrane curvature exerted by the IFITM (72, 73). Dye-dequenching transfer experiments using labelled IAV virions suggest that hemifusion, the mixing of lipids from the outer leaflets of viral and cellular membranes, still occurs in the presence of the IFITM (70, 72). However, whether this is generalizable to all enveloped virions is not known.

One related mechanism, suggested by Amini-Bavil-Olyaee et al., is that the direct interaction of IFITM3 with vesicle membrane protein associated protein A (VAPA) leads to a disruption of the VAPA-oxysterol binding protein (OSBP) function that acts to regulate intracellular cholesterol homeostasis (69). In the presence of IFITM3, endosomal membranes become cholesterol laden, less fluid and functionally impaired, thus blocking viral entry. However, other studies have failed to replicate the latter observation (70), and the lack of VAPA interaction with IFITM1 or 2 is difficult to reconcile with their antiviral properties. Lastly, a recent study has suggested that a ubiquitous zinc metalloprotease, ZMPSTE24, previously implicated in processing nuclear lamins, is an essential cofactor for IFITMs independent of its catalytic activity (76). As yet, the mechanism for its role is not known.

### Restriction of HIV by IFITMs

**Figure 2:**
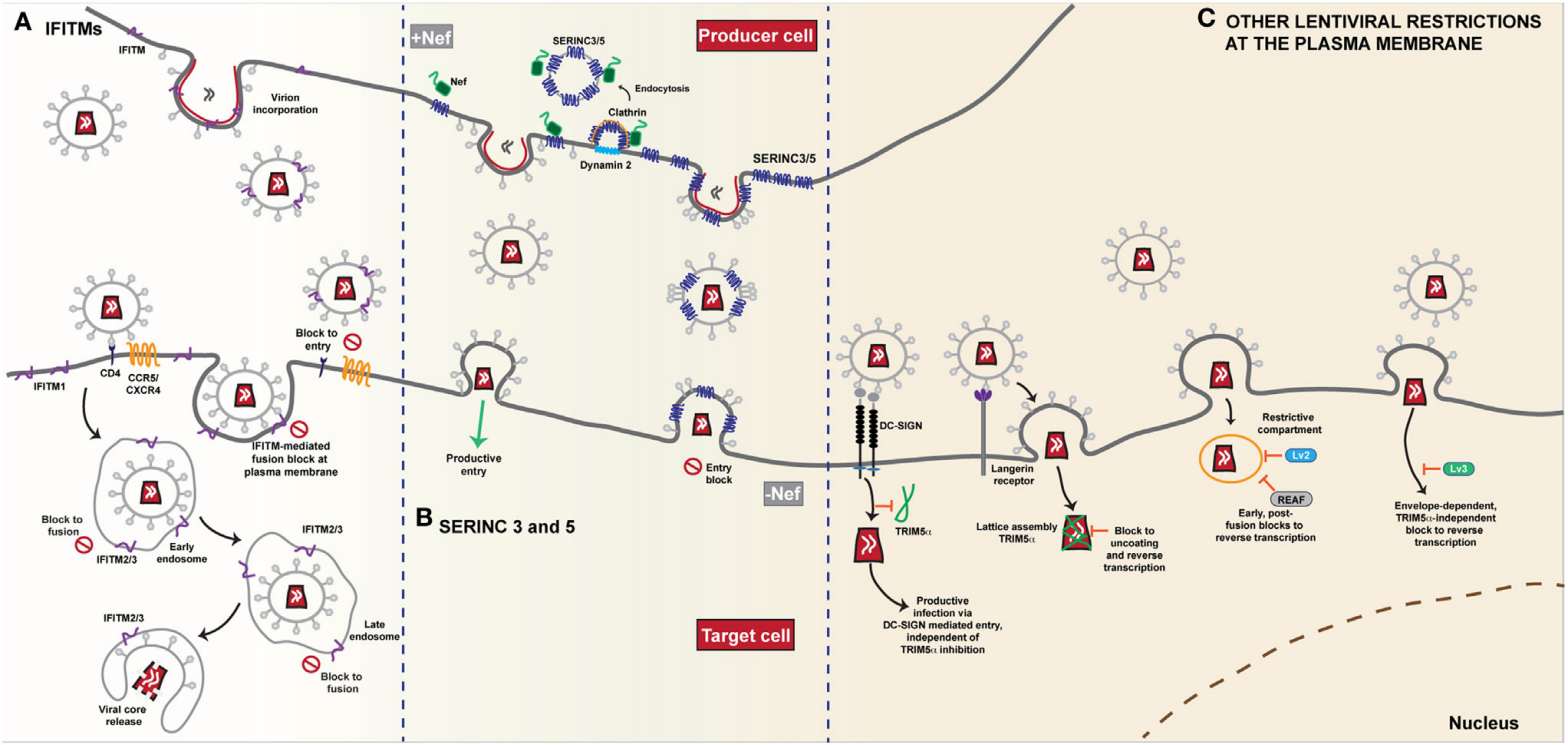
Restriction of HIV-1 entry at the plasma membrane. **(A)** IFITMs. The antiviral restriction activity of the interferon induced transmembrane (IFITM) protein family appears to be linked to the site of viral fusion. The current general mechanism of action proposed, i.e. the physical fusion of the viral and host cell membranes is blocked, accounts for the diversity of viruses that IFITMs restrict. The influence of complex cellular trafficking pathways on this mechanism is yet to be determined. IFITMs 1, 2 and 3 are localised in different membrane compartments; IFITM1 primarily at the plasma membrane and IFITMs 2 and 3 in overlapping intracellular endocytic compartments-the sites of enveloped virus fusion. HIV-1 entry requires the CD4 receptor and co-receptors CCR5 or CXCR4 and it appears that HIV-1 sensitivity to IFITM restriction is influenced both by IFITM localisation and the site of fusion. Fusion that occurs at the plasma membrane is susceptible particularly to an IFITM1 mediated block. IFITMs 2 and 3 appear to restrict any fusion events that bypass the plasma membrane and occur within the intracellular compartments. IFITMs incorporated into viral particles during budding mediate their restriction on the target cell as the virus progeny become increasingly less infectious due to IFITM incorporation. **(B)** SERINC 3 and 5. The transmembrane proteins SERINC3 and SERINC5 are incorporated into budding HIV-1 particles from the membrane of the infected cell. In the absence of the Nef protein, HIV-1 infectivity in the target cell is restricted as delivery of the viral core is reduced due to a block to fusion. Conversely, in the presence of Nef, SERINC3/5 are relocalised from the plasma membrane through dynamin- and clathrin-dependent endocytosis, thus restoring viral infectivity and allowing for successful fusion of the progeny virions that lack SERINC3/5, with the target cell. **(C)** Other lentiviral restrictions at the plasma membrane. The post-entry restriction activity of lentivirus susceptibility factors 2 and 3 (Lv2/3) is dependent on the fusion events at the plasma membrane. Both envelope and capsid are determinants of Lv2 mediated restriction that blocks reverse transcription and nuclear entry. Likewise, RNA-associated early stage antiviral factor (REAF) which has been identified as a potent effector of Lv2, blocks reverse transcription in a similar manner dependent on the route of entry. The Lv3 block is a TRIM5α-independent process that is dependent on envelope interactions with viral entry receptors. The cell specific restriction factor TRIM5α, binds to capsid and forms a lattice leading to premature disassembly of the core. In Langerhans cells, HIV-1 uptake by the C-type lectin Langerin leads to recruitment of TRIM5α and a post-fusion block that occurs prior to integration. Conversely, in other DC subsets, interaction with DC-SIGN, induces a signalling cascade that facilitates reverse transcription and prevents TRIM5α restriction.

All three IFITM proteins have been demonstrated to affect HIV-1 entry and replication, albeit to a lesser degree compared to their effects on other viruses. However, there has been some controversy over their potency and mode of action. The initial study from the Liang group, based on T cell lines ectopically expressing individual doxycycline-inducible IFITMs showed that IFITM2 and IFITM3 could block the entry of a model X4-using laboratory strain, but all three IFITMs could block spreading replication, suggesting multiple stages of the HIV-1 replication cycle were sensitive to IFITM restriction (53). While these differential effects on HIV-1 entry and replication were observed in the target cells, two further studies explored the role of IFITMs in HIV-1 producer cells (77, 78). Both groups observed that IFITMs were incorporated into viral particles, making the particles less infectious. They hypothesized that through cell-cell transmission, the virions were able to circumvent the effect of IFITMs in target cells but cell-free virus spread from infected producer cells is limited as the virions produced become increasingly less infectious through IFITM incorporation (Figure 2A). While IFITM3 was found to accumulate at sites of viral assembly on the PM, neither study reported a specific interaction with the envelope glycoprotein or an effect on envelope density due to IFITM incorporation. A third study reported that IFITM overexpression caused an infectivity defect to virions not because of their incorporation *per se*, but because they appeared to directly interact with nascent gp160 and block its processing to its mature subunits (79). The major caveat to all these studies is that the majority of the mechanistic data are based on un-physiological overexpression mediated either by transient transfection or drug-induction. Whilst all the studies performed RNAi-mediated depletion of IFITM expression levels (which is challenging because of high homology between the IFITMs) to show that a prototypical HIV-1 isolate replicates better in target cells, that this phenotype is because of the mechanisms proposed is unclear. In particular, the block to gp160 processing has not been reproduced by others under more physiological IFITM expression levels (65, 80). However, virion incorporation of IFITMs as a mechanism of reducing viral infectivity has been suggested for diverse enveloped viruses (81).

The subcellular site at which HIV-1 enters has been controversial. Recently, we wondered whether IFITM-mediated restriction might shed light on this controversy (80). Using a panel of model cell lines based on the neuroblastoma cell line U87-MG (long used in HIV-1 entry studies because they express no CD4 or endogenous major coreceptors), we expressed individual IFITMs at interferon-induced expression levels alongside CD4 and CXCR4 or CCR5. We found that IFITM restriction of HIV-1 was mediated by all three proteins but that there was a dependence on the viral co-receptor usage (Figure 2A). Virions that required the CCR5 co-receptor were more susceptible to inhibition by IFITM1 at the plasma membrane whilst CXCR4-using virions were more sensitive to IFITMs 2 and 3 that are predominantly localised within endosomal compartments. We therefore hypothesized that both properties of the viral envelope and that of the IFITM, in particular its subcellular localisation, dictated this “specificity” of inhibition. We showed that mutation of Y19/Y20 that mislocalises IFITMs 2 and 3 to the plasma membrane, or direct blockade of endocytosis, also modulates the restriction activity of these proteins against HIV-1 virus isolates that differ in their sensitivity to restriction by IFITM1 or IFITMs 2 and 3. HIV-1 envelope glycoproteins that were usually sensitive to restriction by IFITMs 2 and 3 were now insensitive in both one-round entry assays and spreading replication. The observation that this did not impair virion incorporation of the IFITM indicated that the primary mode of restriction was the blocking of viral entry by the IFITM expressed on the target cell membrane. These data implied that the pattern of IFITM-mediated restriction of a given envelope indicated different sites of entry—some viruses may fuse at the PM; others in, or en route to, endosomal compartments (Figure 2A). Three independent studies have also linked coreceptor use and IFITM sensitivity [(82, 83) #870] (81). In particular, Huang and colleagues (83) identified a putative splice variant (Δ20 IFITM2) of IFITM2 that lacks the N-terminal 20 amino acids of the full-length protein. They report higher endogenous expression of this isoform in monocytes and in CD4+ T-cells compared to the full-length protein, with localisation of the variant both at the plasma membrane and in endosomal compartments. They found that several R5-tropic viruses were resistant to inhibition by Δ20 IFITM2 with the cytoplasmic tail of CCR5, containing the major trafficking and signaling motifs, being a major determinant of this resistance. By contrast a diverse range of X4-tropic viruses were highly susceptible to inhibition. Whilst confirming that coreceptor usage also affected sensitivities to full length IFITM2 and IFITM3-mediated entry restriction, they found this was cell-type dependent, further highlighting the complexities of IFITM-mediated restriction of HIV entry. Interestingly, the authors showed that IFITM2 knockdown in primary dendritic cells led to a 2-fold increase in their permissivity to X4 viruses. Whilst it is unclear if this was a significant gain in replication capacity for myeloid cells, it raises the possibility that X4 viruses might lack macrophage tropism in part through active host restrictions that R5 viruses avoid, something previously suggested by Schmidtmeyerova et al. 20 years ago (84).

### The importance of IFITM-Mediated Restriction in Transmission and Acute Infection

Amongst the viruses tested in our study, we showed that envelopes from R5-tropic TF viruses were uniformally resistant to IFITM restriction (80). Intriguingly, matched virus clones representing the majority species from the same individual at 6 months had gained substantial sensitivities to IFITM2 and 3 in particular. Again this was envelope determined, and was lost upon relocalization of the IFITM to the PM, suggesting that changes in Env during those 6 months had affected the route of viral entry, despite no change in coreceptor usage occurring. A major determinant of the IFITM-resistance of the TF virus was the cell surface level of CD4, suggesting that receptor engagement and density were key requirements. Consistent with this idea, selection of X4 HIV-1 resistance to IFITM1 by the Liang group yielded viruses with lesions in Vpu and changes in the CD4 binding site of Env (85). Such adaptations in culture will lead to a modulation of the envelope structure during assembly (see tetherin section below). The differences in Env between TF and 6 month viruses varied between individuals. It is well known that gp120 and gp41 are the targets for both T cell and antibody responses throughout infection *in vivo*. Hypothesizing that escape from such adaptive immune responses in Env might reveal IFITM sensitivity, we found that reversal of amino-acid changes in gp120 that arose through the escape of early neutralizing antibody responses (86) fully restored IFITM resistance to the 6-month virus. Furthermore, in primary human CD4+ T cells, knockdown of IFITM2 and 3 rescued much of the 6 month virus’s replication after IFN-treatment. Thus it would appear that IFITM-resistance in Env is a major contributor to the overall IFN-I resistance of transmitted viruses, implying their evasion must be an important attribute for successful transmission. Moreover, once the virus is systemically established, structural changes in Env that affect receptor/co-receptor interactions leading to IFITM sensitivity become tolerable if there is selective pressure applied by a competing adaptive immune response. This suggests that even host restrictions with a relatively small magnitude [by comparison to say APOBEC3G (87)] can have a major determining effect at transmission or in the early stages of systemic replication. Furthermore, because IFITM sensitivity appears to be dictated by Env/receptor interactions, these data further suggest a constraint on the envelope at transmission that endows it with IFN resistance, which itself may be an important consideration for vaccine design. Of note, recent studies on the adaptation of chimeric SIVs encoding HIV-1 envelopes (SHIVs) via sequential passage in macaques demonstrated a gain in IFN resistance mapping to Env, and particularly its level of virion incorporation (88, 89). Whether this is reflective of restriction by simian IFITMs, which do inhibit lentiviruses in culture (90), has yet to be determined.

The *ifitm* locus is complex and has not been well-annotated for genome wide association studies. However, SNPs in *ifitm3* have been implicated in the susceptibility to human disease. Of these, *rs12252* has generated much interest. Homozygosity for a very rare minor allele, *rs12252-C*, was strongly associated with the severity of H1N1 Swine Flu in the UK (91). This synonymous polymorphism changes a serine codon in the N-terminal cytoplasmic tail of IFITM3 from AG**T** to AG**C**. Initially, this was thought to lead to an alternatively spliced message that would express a N-terminally truncated IFITM3 protein lacking its endocytic YXXϕ motif. Such a truncated protein localizes to the PM and does not restrict IAV entry (91). However, no evidence of such a splice variant has since been found, raising questions about how this SNP exerts its effects. Reproduction of *rs12252-C* association with IAV pathogenesis has been mixed, but in Han Chinese populations, where the allele frequency is much higher (30–40%), a clear association with flu severity has been confirmed (92–97). At present it is not known whether other SNPs in the locus are in linkage disequilibrium with *rs12252-C* that might explain such discrepancies. In the same Chinese population *rs12252-C* is also strongly associated with rapid progression during acute HIV-1 infection, and in particular elevated viral loads and CD4+ T cell loss (96). Unlike IAV pathogenesis, this association was also observed in heterozygotes, suggesting the effect of *rs12252-C* is dominant. These intriguing results further highlight the importance the IFITMs in HIV-1 pathophysiology. The elucidation of the molecular bases for these observations will provide mechanistic insight to their role in HIV restriction.

## Serine Incorporators 3 and 5

The accessory protein Nef, common to all primate lentiviruses, has a multitude of functions in HIV-1 replication (42). Nef is myristoylated and associates with the inner leaflet of the PM and endosomal membranes. Here it promotes downregulation of various membrane proteins from the cell surface, predominantly to reduce the recognition of infected cells by adaptive immune responses. The most well-studied Nef targets are CD4, and class I and II MHC molecules, which protect infected cells from antibody-dependent cellular cytotoxicity (ADCC) (98) or recognition by antigen-specific T cells respectively, although several others have been identified (42), particularly amongst SIV Nef alleles. However, one conserved function of lentiviral Nef proteins that until recently remained unexplained, was its ability to promote the infectivity of the lentiviral virion (99).

Cells infected with HIV-1 mutants lacking Nef produce virions with reduced infectivity, even in the absence CD4 which itself interferes with envelope folding and trafficking (99). The magnitude of this CD4-independent effect on virion infectivity is variable amongst cell lines, but from lymphoid cells it can be reduced by as much as 50-fold (6). Pseudotyping virions with heterologous pH-dependent envelope proteins such as the glycoproteins from vesicular stomatitis virus or Ebola virus completely rescues the infectivity defect of HIV-1 Nef mutants (99). However, while this infectivity defect is manifest at an early entry or post-entry stage, it does not correlate with envelope incorporation into the virion. Furthermore, variations in gp120 variable domains, particularly the V1/V2 loops, affect the sensitivity of HIV-1 to Nef-dependent infectivity enhancement, implying that Nef regulates an intrinsic property of Env during the entry process (100). In keeping with this, Nef also affects the sensitivity of virions to certain neutralizing antibodies (101).

The first clue that this may be governed by a host restriction factor came from the observation that Nef interaction with dynamin 2 (dyn2), the major cellular GTPase that controls endocytosis, was essential to regulate particle infectivity (102). The requirement for dyn2 by Nef was during viral production, and its knockdown reduced virion infectivity to that of the Nef-defective mutant. Since Nef mediates the removal of other membrane proteins from the cell surface, one attractive hypothesis was that it was targeting an inhibitor of virion infectivity. This was further evidenced by the demonstration that in heterokaryons between human cells that had a high and low dependence on Nef for virion infectivity, the requirement for Nef was dominant (6). Intriguingly, the accessory protein of gamma retroviruses, a membrane-bound and glycosylated form of their major structural protein Gag (GlycoGag), can substitute for Nef activity and vice versa (103). GlycoGag is generated from a weak in-frame translational start site upstream of the regular Gag initiation codon, producing a Gag with an 88 amino acid N-terminal extension that results in its insertion in the ER membrane. As with Nef, GlycoGag promotes MLV infectivity in a dyn2 and endocytosis dependent manner (104), thus indicating that they target a common factor or pathway.

In 2015 two groups cloned the factor(s) responsible for this phenotype by complementary approaches. In the first, Massimo Pizzato and colleagues performed a large scale gene expression analysis of cells where the virus dependence on Nef varied, looking for mRNAs whose abundance correlated with the magnitude of the infectivity enhancement (6). In the second, Heinrich Gottlinger’s group performed proteomic analyses of HIV-1 virions purified from human T cells in the presence or absence of Nef and/or GlycoGag expression, hypothesizing that a Nef-regulated inhibitor of infectivity may be incorporated into virions of Nef-defective viruses (8). Both groups identified members of the serine incorporator (SERINC) family of multi-pass membrane transporters, SERINC5 and SERINC3 respectively. Shortly afterward, a further proteomic study documenting global changes to the cell surface proteome of HIV-1 infected T cell lines identified both SERINCs as differentially regulated by wild-type and Nef-defective viruses (105). Both proteins were shown to inhibit Nef-defective virus infectivity upon ectopic expression in “low Nef-responsive” cells, with SERINC5 being the most potent (6, 8, 106). Conversely, CRISPR/Cas9 knockout of both SERINC5 and SERINC3 fully restored Nef-defective virus infectivity from CD4+ T cells. In the presence of Nef, SERINC5 is relocalized from the PM to endosomal compartments dependent on Nef interaction with the clathrin adaptor AP-2 (Figure 2B). Moreover, SERINC5 was also counteracted by various SIV Nef alleles as well as MLV GlycoGag and VSV-G (6, 8), thus recapitulating the known features of the proposed restriction factor. Additionally, the S2 accessory protein of the distantly related lentivirus, equine infectious anemia virus (EIAV), also counteracts SERINC5 (107). Interestingly, unlike the IFITMs or tetherin (see below), SERINCs are neither significantly regulated by IFN-I, nor do they display evidence of positive selection in mammals (6, 108).

At the time of writing almost nothing is known about the mechanism by which SERINC5 exerts its antiviral activity. SERINCs are PM proteins with 12 predicted TM domains (Figure 1B). They are conserved from yeast to man, but only SERINCs 3 and 5 restrict retroviral infectivity (6, 8). Whilst there are several predicted isoforms of SERINC5 derived from putative splice variants, the majority mRNA species encodes the longest form (109). SERINCs were originally named for their proposed ability to incorporate serine into membranes as phosphatidylserine or sphingolipids (110), although how they do this or even whether this activity is relevant for viral restriction is not known. Direct incorporation of SERINC5 into the virion seems to be essential, and as a result of Nef-mediated internalization, SERINC5 is excluded from the assembling virion (6, 8, 111). However, this is not sufficient to explain the antiviral activity as VSV-G pseudotyping of the virus confers complete SERINC5 resistance without blocking incorporation (6, 8). What has been shown is that the block mediated by SERINC5 occurs at the fusion stage (6, 8). Both particle-associated beta-lactamase (BLAM) or CRE recombinase transfer to target cells is reduced in the presence of SERINC5, however the magnitude of this block to fusion does not fully match that of the infectivity defect or levels of reverse transcription. Whilst this has been interpreted as a potential block to fusion pore expansion rather than the initiation of fusion, it could also simply be a reflection of the difference in the dynamic range of assays that measure entry and post-entry events. Interestingly, SERINC5 sensitivity of primary R5 tropic viruses is variable in the absence of Nef (8). Exchange of the gp120 V1/V2 or V3 loops between these and prototypic X4 viruses swaps these phenotypes. This in part maps to variable N-linked-glycosylation sites in gp120 that are thought to stabilize the envelope glycoprotein (100). A very recent study indicates that while there is no evidence yet of direct Env/SERINC5 interaction, sensitive envelopes appear to be inactivated, exposing epitopes that would normally require receptor interactions (112, 113). Thus SERINC5 may be affecting the intrinsic stability of the Env trimer, thus blocking fusion. It is interesting to note the potential parallels here with those of the restriction of HIV-1 by IFITMs, with the relative resistance of R5 envelopes again highlighting that constraints on the envelope glycoprotein may be driven by selection for their resistance to intrinsic antiviral restriction mechanisms.

As noted above, SERINC3/5 expression appear not to be regulated by inflammatory stimuli and there is no evidence of the positive selection in mammalian SERINCs that is a common feature of other viral restriction factors (6). There is no apparent species specificity in antagonism, with a given HIV-1, HIV-2 or SIV Nef counteracting both human and primate SERINC5 orthologues (114). This conservation of function in Nef would in itself imply its importance. However, further observations have hinted that the efficiency of Nef-mediated SERINC antagonism by HIV and SIV Nef alleles may correlate with prevalence of a given virus in its host primate species (114). If so, then the selective pressure on Nef that gives rise to this variation in activity will be more complex than simply Nef/SERINC5 interaction, and may reflect, for example, impacts of envelope variation in SIVs or other properties of SERINCs in lentiviral replication yet to be discovered.

## Other Lentiviral “Route of Entry” Restrictions

Aside from IFITMs and SERINCs, other restrictions have been reported that affect post-entry events in lentiviral replication dependent on the route of viral entry. These restrictions, termed Lv2 and Lv3 [Lv1 being the name of the post-entry restriction activity later shown to be conferred by species-specific variants of TRIM5α (115, 116)], operate in human and primate cells respectively. Lv2 manifests as a block to reverse transcription and nuclear entry, and was originally demonstrated for HIV-2 in certain human cell lines and primary macrophages (Figure 2C) (117). The viral determinants of this restriction mapped both to the viral capsid and envelope proteins, but the entire restriction can be bypassed by VSV-G, suggesting that the post-entry block depends on where in the cell the virus fuses (118). Consistent with this, Lv2 restriction can be relieved by blocking endocytosis or mis-localizing CD4 at the PM (119). Moreover, these restriction patterns can also be seen for a variety of X4-using HIV-1 strains and be in part conferred to a heterologous core by envelope pseudotyping (119), a phenotype that bears some similarity to those for IFITM-mediated restriction (80). However, more recently regulation of nuclear pre-mRNA domain-containing protein 2 (RPRD2), termed by the authors REAF (RNA-associated early stage antiviral factor), has been proposed to be the effector of Lv2 restriction (120, 121). REAF appears to interact with the incoming genome to block reverse transcription, but is dependent on the pseudotyping envelope (Figure 2C). Whether REAF is differentially localized along the endocytic network, or whether restrictions during fusion (or avoidance thereof) predispose the incoming virus to REAF-mediated restriction remains to be determined. Similarly, Lv3 is a post-entry block to HIV replication in macaque cells that is distinct from TRIM5α and again depends on the Env CD4/coreceptor interactions (122). Again, this bears superficial similarities to IFITM restrictions, but the block appears to be manifest at reverse transcription and can be saturated (Figure 2C).

A third very recent example of “route of entry restrictions” has been described in a human dendritic cell subset, Langerhans cells (LCs), that are resistant to HIV-1 infection due to the interaction between the virus and the C-type lectin langerin (123). This mediates virion targeting to Birkbeck granules and prevents viral replication at an early post-entry stage. This turns out to be dependent on human TRIM5α, the capsid-binding restriction factor to which HIV-1 was thought to be resistant. In the presence of langerin, the authors propose that TRIM5α is recruited to the site of entry and targets the incoming virus to an autophagic degradation pathway. In other DC subsets, virion engagement with a different lectin, DC-SIGN, prevents the recruitment of TRIM5α upon virion internalization (Figure 2C).

Type II IFNs (IFNγ) have an under-appreciated direct antiviral activity on HIV-1 (124). In part, this again maps to the envelope protein, and in particular the V1/V2 loop (124). The initial results suggest that TF viruses may be more resistant to the effects of IFNγ, but the factors involved are not yet known.

## Tetherin

At the other end of the viral lifecycle, the most prominent antiviral inhibitor of lentiviral replication associated with the plasma membrane is tetherin (also known as bone marrow stromal cell antigen 2—BST2 or CD317). Tetherin’s antiviral activity was discovered as the target of the HIV-1 accessory protein Vpu (125, 126), long known to play a role in the efficient release of new retroviral particles from infected cells. Tetherin is an IFN- and pattern recognition-regulated gene and has a general antiviral function against diverse enveloped viruses [reviewed in Ref. (5)]. Amongst the primate lentiviruses, tetherin antagonism is a highly conserved attribute (127). Furthermore, the adaptation of HIV-1 Vpu to target the human tetherin orthologue was a key event in the development of the HIV/AIDS pandemic. In this section we will focus only on the role of tetherin in lentiviral pathogenesis.

### Tetherin-Mediated Restriction of Viral Release

**Figure 3:**
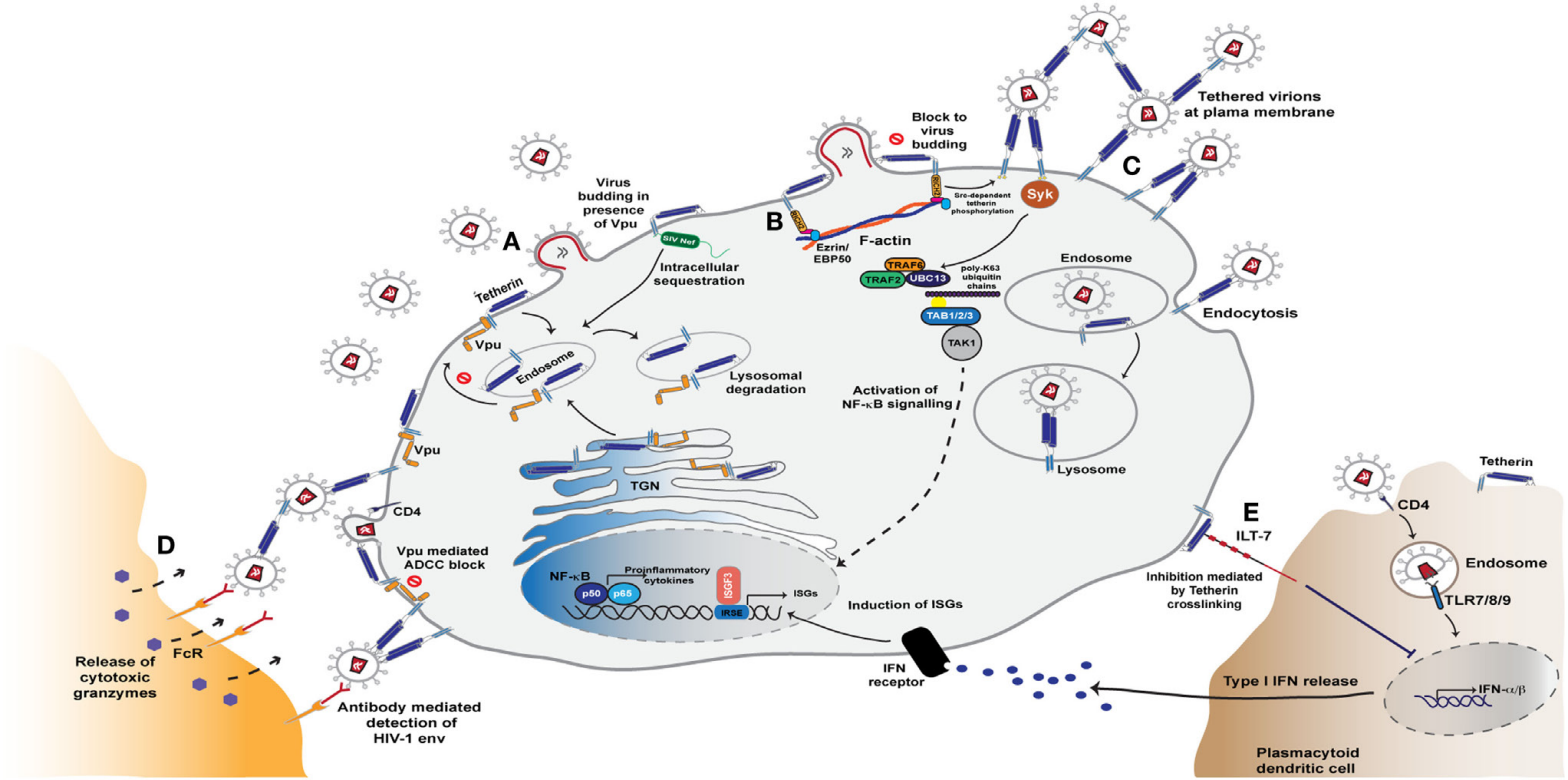
Tetherin mediated restriction of HIV-1 assembly and release. **(A)** Tetherin resides in the plasma membrane and is constitutively recycled from the plasma membrane via endosomes and the trans-Golgi network (TGN). The antiviral activity of tetherin is counteracted by the Vpu and Nef proteins. During HIV-1 infection, Vpu binds to the transmembrane domain of tetherin in the endoplasmic reticulum and Golgi network and sequesters it in endosomal regions in a clathrin-dependent manner. This rerouting of tetherin from the plasma membrane leads to its degradation in lysosomes via the ESCRT pathway. SIV Nef, too, is able to antagonise tetherin activity by sequestering tetherin from the sites of virus assembly, leading to lysosomal degradation of the entrapped molecules. **(B)** In the absence of Vpu and Nef activity, tetherin accumulates at virus assembly sites and blocks virus release from the plasma membrane. This clustering of retained virions triggers a signalling cascade mediated by tetherin’s cytoplasmic tail that leads to NF-κB activation and release of proinflammatory cytokines. This sensing of retention is reliant on tetherin association with the actin cytoskeleton via the adaptor protein RICH2. **(C)** Signalling that ultimately leads to NF-κB activation triggered by the phosphorylation of tetherin monomers and recruitment of the Syk kinase. This in turn leads to recruitment of the E3 ligases TRAF2 and TRAF6, that with the E2 enzyme UBC13, and K63-ubiquitin-mediated activation of the kinase TAK1 to activate NF-κB **(D)** Virions tethered at the cell surface are also exposed to anti-Env antibodies that thereby sensitise the infected cell to anti-HIV antibody-dependent cell-mediated cytotoxicity (ADCC) responses from Fc-receptor expressing myeloid and NK cells. **(E)** Tetherin is able to further modulate the cell’s innate immunity through activation of the plasmacytoid dendritic cell specific leukocyte inhibitory receptor ILT7. Interaction with this ligand results in dampened TLR signals that thereby decrease type I interferon (IFN-I) production and enhancement of immune responses.

Tetherin is a type 2 membrane protein whose distinctive topology is indicative of its primary mode of action: the retention of fully-formed virions on the PM of infected cells and their subsequent removal to endosomes (128, 129). Tetherin exists in the PM as disulfide-linked dimers that constitutively recycle via the Golgi apparatus (130, 131). The extracellular domain of tetherin forms a rod-like coiled-coil, with a hinge towards its N-terminal transmembrane domain to allow a degree of rotational flexibility (Figure 1C) (132–134). The C-terminus is covalently attached to the lipid of the PM by a glycophosphatidyl-inositol (GPI) linkage, giving the mature protein two membrane anchors (Figure 1C) (128, 130). As the nascent virus buds through the PM, tetherin dimers are recruited to the virion membrane (128, 135, 136). The C-terminal GPI anchor appears to be preferentially incorporated into the virion whilst the N-terminal TM domain is retained outside the bud (Figure 3) (129). When the ESCRT pathway mediates the scission of viral and cellular membranes, tetherin dimers retain the new viral particle via a stable protease-sensitive crosslink (125, 129, 137, 138). Leaky scanning of the tetherin mRNA leads to two isoforms being expressed at apparently equal levels, differing in the length of their cytoplasmic tails (139). Depending on the species orthologue, the shorter isoform lacks the first 12–17 amino acids that encompass the major subcellular trafficking signal—a dual tyrosine-based motif that engages clathrin adaptors AP1 and AP2 (131). Both isoforms can form homo- and heterodimers and both can potently restrict viral release (139, 140). However, the longer human isoform has a proinflammatory signalling activity associated with it (see below), and is also more sensitive to Vpu (139, 140).

Tetherin expression is induced by both type I and II IFNs, as well as pattern recognition signals, in many cell types (141, 142). It is expressed on activated T cells and is constitutively expressed by plasmacytoid dendritic cells. Tetherin expression is upregulated on peripheral blood mononuclear cells during the acute phase of HIV infection (143), and by treatment of HIV-infected individuals with IFNα (45). Its expression is enriched on tissues with barrier function, further suggesting an important role in host defence (144).

### Tetherin Counteraction by Primate Lentiviruses and Its Role in Limiting Cross-Species Transmission

Tetherin targets a part of the virus that it cannot mutate to evade restriction, therefore the virus must evolve a countermeasure. Although the virally-encoded protagonist and mechanism differ, the ability to counteract tetherin is conserved among primate lentiviruses (5).

SIVs are naturally prevalent in a wide range of African non-human primates [reviewed in Ref. (145)]. For the most part each species is infected with a monophyletic strain of SIV (indicated by a suffix denoting the host species e.g. SIVsmm in sooty mangabeys), signifying predominantly within-species spread, with some notable examples of cross-species transmissions. Over 40 primate lentiviruses have been identified, and of these three have crossed the species barrier into humans: SIVcpz, SIVgor and SIVsmm, from chimpanzees, gorillas and sooty mangabeys respectively (145).

The precursors to HIV-1 were transmitted from chimpanzees to humans on at least 2 separate occasions, giving rise to HIV-1 groups M and N (146, 147), and twice from gorillas to humans resulting in HIV-1 groups O and P (148, 149). The precursors to HIV-2 crossed from sooty mangabeys into humans at least 8 different times (or at least their sequence diversity suggests independent cross-transmissions), resulting in HIV-2 groups A-H (150–152). These 12 groups of viruses have had vastly different impacts on the human population, ranging from single-case HIV-2 infections to the millions of people infected with Group M since its first predicted zoonotic infection in the early 1900s (145). While environmental and social factors inevitably played a role in the outcome of these zoonoses, extensive work dissecting host-pathogen relationships reveals a role for tetherin in influencing the course of cross-species infections.

Most SIVs counteract their host’s tetherin using the accessory protein Nef (127, 153, 154). Notable exceptions to this are SIVs from greater spot-nosed, mustached and mona monkeys (SIVgsn, mus and mon respectively) which are unique among SIVs in possessing the accessory protein Vpu, capable of antagonising tetherin in a species-specific manner (127). Although SIVcpzPtt and SIVgor are also among the subset of SIVs that possess a *vpu* gene, their Vpus lack the ability to counteract chimpanzee, gorilla and human tetherin, although they still maintain function in the form of robust CD4 downregulation (127). These viruses use Nef as an antagonist, which stimulates the AP2-dependent clathrin-mediated endocytosis of tetherin, removing it from the site of virus assembly. The use of Nef rather than Vpu as a tetherin antagonist may be explained by the origins of SIVcpz—a chimaeric virus originating from recombination between an ancestral strain of the SIVgsn/mus/mon lineage and red-capped mangabey SIV (SIVrcm) (155). Inheriting two tetherin antagonists appears to have resulted in SIVcpz losing counteractivity in one.

The deletion of a five amino acid stretch (G/DIWKK) in the cytoplasmic tail of tetherin (Figure 3) between 1 and 6 million years ago—after divergence from chimpanzees but before the divergence of Denisovans and Neanderthals—has rendered the human protein resistant to SIV Nef antagonism (127, 153, 154, 156). Consequently, establishing a successful infection in humans requires an alternative mechanism of tetherin counteraction, either by adapting a different antagonist or adjusting the action of Nef. As detailed below, the mechanism and/or the extent of the adaptation differs in each known case of cross-species transmission.

HIV-1 group M Vpu efficiently deals with both tetherin’s physical virus restriction and subsequent antiviral signalling by escorting nascent tetherin into a defunct cellular pathway and triggering its degradation (5). Vpu and tetherin interact via their transmembrane domains, with the interactive face of Vpu consisting of highly conserved alanines and a tryptophan (Figure 3) (157–160). Moreover, it is this interacting face that was likely to have been the key adaptation that led to human tetherin counteraction by the prototypic group M HIV-1 as revealed by the Vpu sequences of its closest extant SIVcpzPTT relatives (161). Tetherin/Vpu complexes are then targeted to late endosomes for degradation (162). This complex process requires the phosphorylation of the Vpu cytoplasmic tail that facilitates the formation of a ternary complex between tetherin, Vpu and the clathrin adaptor AP-1, and perhaps AP-2, promoting their targeting to late endosomes (163–165). This mechanism allows Vpu to engage both newly synthesized and recycling tetherin pools. Concomitant with this process, the dual-serine phosphorylation site of Vpu, a conserved DSGxxS motif, interacts with an SCF E3 ubiquitin ligase, predominantly through the adaptor protein βTRCP2 (162, 166–169). This leads to multiple ubiquitination events in the Vpu cytoplasmic tail (170–172) that target it for ESCRT-mediated degradation (164, 173–175). This final rerouting and degradation of tetherin requires the major endocytic motif in its cytoplasmic tail (163, 175). Thus the short isoform of tetherin cannot be degraded or downregulated from the surface by Vpu (140). However, physical interaction with Vpu does reduce its incorporation into virions, counteracting tetherin at lower expression levels.

Primary HIV-1 group M Vpus are highly active antagonists of tetherin and efficient inhibitors of tetherin-mediated NF-κB signalling, and these functions are conserved in transmitted viruses and throughout the course of infection, and across the clades (127, 176–178). Suboptimal Vpus are rapidly selected against *in vivo*, and robust anti-tetherin function is maintained even years after infection (177). Studies of viruses with mutations in Vpu rendering them specifically unable to counteract tetherin but otherwise unaffected, demonstrate that these viruses are compromised compared to wildtype viruses in the presence of high concentrations of IFN-I (179). Likewise, selective pressure provided by upregulated tetherin expression during IFNα treatment of HIV-infected individuals may select for changes in Vpu (45). Thus, Vpu-mediated tetherin counteraction contributes to the overall viral interferon resistance.

Interestingly, it appears that Group M Nefs are able to acquire moderate ability to counteract human tetherin in certain circumstances (180). Although this does not represent a common activity amongst Group M Nefs, the association of a proportion of the active Nefs with viruses harbouring defective Vpus further underlines the importance of tetherin antagonism *in vivo* (180).

Fewer than 20 cases of Group N infections have been documented to date, and their adaptation to human tetherin represents a mixed and developing picture. For the most part they display some ability to counteract tetherin and enhance infectious virus release from cells, but activity is poor compared to the typical levels of Group M Vpus (127). However, a highly pathogenic Group N virus isolated from a French individual—the first case of Group N infection found outside Cameroon—demonstrated Vpu activity on a par with that of Group M. This French/Togo Vpu contains functional domains known to contribute to activity in Group M Vpus, whilst these are lacking in other known weak Group N Vpus (181). The mixed success of Group N Vpus to combat human tetherin is counterbalanced by its total inability to perform another major function of Vpu, the downregulation of CD4.

HIV-1 Group O infections represent a substantial epidemic, with an estimated 100,000 people infected. The majority of Group O Vpus tested demonstrate poor tetherin antagonism (127, 182, 183); instead, Group O Nef has adapted to target a different region of human tetherin, circumventing the 5 amino acid deletion that confers resistance to inhibition by SIV Nefs (184). The activity of the Group O Nefs is species-specific, being more efficient at downmodulating human compared to gorilla tetherin. Interestingly, a single example of a Group O Vpu able to counteract tetherin has recently been reported (185).

HIV-1 group P viruses have been isolated from only two individuals to date, both from Cameroon (186, 187). These viruses appear to be poorly adapted to humans, with no tetherin counteractivity detected in either their Vpu, Nef or Env proteins (183, 188).

Like most SIVs, the SIVsmm precursor to HIV-2 uses Nef to antagonise tetherin in its sooty mangabey host (127). Similar to SERINC5 antagonism, SIV Nefs bind to their cognate primate tetherin dependent on the G/DWIKK motif and promote its AP-2-mediated endocytosis from the cell surface (189, 190). While HIV-1 Group M evolved efficient tetherin antagonism by Vpu, and Group O Nefs evolved to target a different region of tetherin (184), HIV-2 employs a different strategy of antagonism, using the Env protein (191). The extracellular domains of both proteins interact, and again this stimulates endocytic removal of tetherin from the cell surface through Env’s interaction with AP-2 (191–194). Tetherin antagonism appears to be a conserved attribute of HIV-2 isolates tested to date (195), although the potency of HIV-2 Env in enhancing virus release is weaker than that of HIV-1 group M Vpus, insofar as *in vitro* assays are a true reflection of activity. Whether there is a fitness and efficacy cost associated with using a major structural protein, also under pressure to evade antibody responses, to carry out a role more commonly performed by accessory proteins remains to be seen.

### *In Vivo* Relevance—Evidence from Experimental Infections

The importance of tetherin *in vivo* is demonstrated by the remarkably diverse strategies enlisted by viruses to overcome this barrier (5). Simple demonstrations of this arms race in action come from experimental infections of primates, of which there are several examples demonstrating pathogenesis associated with acquisition of tetherin counteractivity. Studies of chimpanzees infected with HIV-1 for the purposes of vaccine studies in the 1980s were revisited in order to investigate readaptation to a previous host species. Examination of the readapted viruses revealed that, although the Vpu maintained function, tetherin antagonism was also acquired in Nef, with the virus using both proteins to overcome chimpanzee tetherin (196). The minimal changes required to restore anti-chimpanzee tetherin activity to the HIV-1 Nef were just 2 amino acids, and the region of chimpanzee tetherin targeted by the adapted Nef was mapped to the DIWKK region deleted in human tetherin (196). It therefore appears that lost accessory gene functions can be reacquired relatively easily. Similarly, serial passage of modified simian tropic HIV-1 in pigtail macaques resulted in a virus that could replicate efficiently and cause AIDS in these otherwise unsusceptible hosts (197). The modified virus used in the original inoculum was endowed with resistance to macaque APOBEC3 restriction factors, but unable to counteract monkey tetherins. Four passages resulted in a pathogenic virus that was able to efficiently counteract macaque tetherin while maintaining anti-human tetherin activity. The amino acid changes responsible for this adaptation were mapped to the transmembrane region of the Vpu—the region that interacts with tetherin—and involved only two amino acid changes (197).

Infection of rhesus macaques with Nef-deleted SIV (SIVmacΔnef) usually results in attenuated infection, with persistent but low-level viral replication. After serial passage these viruses can revert to pathogenicity, leading to high viral loads and progression to disease (198). Analyses of the pathogenic revertant viruses confirmed that these viruses had adapted to counteract rhesus tetherin, with determinants mapping to the cytoplasmic tail of the envelope protein gp41. The minimal changes required to endow Env with this Nef-like activity involved just five amino acids (199). Acquisition of tetherin counteraction in SIV envelopes has been documented in the SIVtan envelope, most likely through passage in human cells (200). More recently, such an adaptation has also been observed in an *in vivo* for a highly neurotropic SIVsm (201).

Studies in tetherin knock-out mice provide direct evidence of tetherin’s antiviral role *in vivo*, with increased replication and pathogenicity of a murine retrovirus observed in the absence of tetherin (202). Otherwise normal development of −/− mice, including no detectable adverse effects on the immune system, further support the primary function of tetherin as an antiviral effector protein. Indeed, most mammalian tetherin orthologues possess antiviral activity, and the role of tetherin as an ancient immune effector molecule is supported by the demonstration of identifiable tetherin orthologues with antiviral activity in reptiles and as far back as the coelacanths (203, 204).

### Tetherin’s Role in Linking Innate and Adaptive Immunity

Tethering viruses to the producer cell membranes and preventing their release is an obvious obstruction to virus propagation. However, the major mode of HIV transmission in cultured T cells is via synaptic conjugations between infected and uninfected cells. These virological synapses are driven by Env/CD4 interactions and result in polarized secretion of new virions across the synaptic cleft (205). While very potent at blocking cell free virus release, the inhibitory effects of tetherin on cell-to-cell spread via the virological synapse structures is weak. In primary human CD4+ T cells, Vpu-defective viruses even spread faster due to tetherin-mediated cell-associated virus accumulation, despite lower cell-free virion release (206). Given the high selection pressure to maintain tetherin counteraction in lentiviruses, it has therefore been of particular interest to determine whether the consequences of restriction have wider ramifications than simply the physical prevention of dissemination. Viruses tethered to the cell surface are exposed to anti-Env antibodies, particularly those targeting CD4-induced epitopes, and this sensitizes the infected cell to ADCC-mediated elimination by Fc-receptor bearing myeloid and NK cells (Figure 3) (98, 207–209). This effect is enhanced by treatment of cells with IFNα due to increased tetherin expression. In turn it is effectively suppressed by HIV-1 Vpu and Nef, which play dual roles by counteracting tetherin and by degrading CD4, therefore protecting the nascent Env trimers from exposing CD4-dependent epitopes and reducing the numbers of cell-associated virions (98, 207–209). Importantly, tetherin therefore acts as a link between innate and adaptive immunity, enhancing the potency of antiviral antibodies and increasing the pressure on the virus to maintain efficient tetherin antagonism.

The clustering of cell surface tetherin molecules due to virus retention triggers signalling events mediated by its cytoplasmic tail, leading to NF-κB activation and the release of pro-inflammatory cytokines (139, 210, 211). These cytokines could potentially serve to further amplify tetherin’s role in ADCC by recruiting effector cells to the site of infection. Tetherin’s signaling activity is restricted to homodimers of the long isoform (139). In this context the major endocytic site, a dual tyrosine motif YDYCRV, acts as a hemi-immuno-tyrosine activation motif (212). Upon virion retention, tyrosines on both L-tetherin monomers become phosphorylated by Src-family kinases and present an SH2-domain for the recruitment of the kinase Syk (212). This in turn recruits a signaling complex including TRAF2, TRAF6 and TAK1, ultimately activating NF-κB (Figure 3) (211, 212). Thus in addition to retaining virions at the cell surface, tetherin acts akin to a pattern recognition receptor in sensing virus restriction. This sensing is dependent on tetherin’s link to the cortical actin cytoskeleton via an adaptor protein RICH2 (AHRGAP44) (212, 213). There appears to be some primate species specificity in tetherin’s signaling activity. The deletion that occurred in chimpanzee tetherin that rendered the human orthologue resistant to Nef antagonism, and serves as a highly effective barrier to cross-species transmissions, also appears to have contributed to the efficiency with which human tetherin initiates proinflammatory signalling (210). In human cells this correlates with primate tetherin phosphorylation efficiency and Syk recruitment (212). Whether this is truly an neofunctionalization of tetherin during primate evolution, or reflects species incompatibilities in experimental cellular systems is not clear. However, in mice knocked-in for constitutive somatic human tetherin expression, runting and early lethality is observed consistent with chronic inflammatory signaling (214).

A further intriguing link between tetherin and innate sensing of viruses is its identification as a ligand for the leukocyte inhibitory receptor, ILT7, expressed on plasmacytoid dendritic cells (pDCs). Interaction between tetherin and ILT7 induces an inhibitory signal that dampens responses by TLR ligands (Figure 3) (215). Recent data from the Cohen group suggests that the ILT7/tetherin interaction acts akin to a ‘missing self’ signal when a pDC encounters a cell infected with a tetherin-sensitive virus (216). The recruitment of tetherin into budding virions occludes its ability to interact with ILT7 on the pDC, thereby enhancing the responsiveness of the pDC if simultaneously encountering extracellular RNA. The authors postulate that differential surface removal of long and short tetherin isoforms by HIV-1 group M Vpu (and some extent Group O Nefs) ensures a sufficient pool tetherin at the PM to deliver this inhibitory signal at the same time as counteracting its antiviral effects (216, 217). Whether this is a universal function of tetherin is unclear; mice lack an ILT7, and a functional orthologue has yet to be identified. However, the upregulation of tetherin on some cancers may suggest that ILT7 interaction is important for tumor-cell immune evasion (215).

Together these observations indicate that tetherin’s antiviral activity *in vivo* is not limited to the physical reduction in cell free virus produced from the infected cell. Rather, virion-tethering to the cell has important knock-on effects on how it is perceived and dealt with by both the innate and adaptive immune response. This linkage between direct antiviral activity and the augmentation of downstream immune responses would thus further explain the high level of selective pressure on viruses such as HIV-1 not only to counteract tetherin for efficient transmission, but to maintain this activity after the establishment of systemic infection where the physical impairment of viral release has only minor effects on spread to new target cells.

## Other Inhibitors of HIV-1 Release and Assembly at the Plasma Membrane

Whilst the most prominent, tetherin is unlikely to be the only antiviral factor that targets HIV during the assembly and release stage. In principle many adhesion molecules or lectins could exert an antiviral effect on virus release provided they, or their ligand, are incorporated into viral particles. Indeed, in the absence of both Vpu and Nef, CD4/Env interactions can limit HIV release (218) as well as exposing epitopes for ADCC.

The T-cell immunoglobulin and mucin domain (TIM) family of phosphatidylserine (PS) receptors have been implicated as important attachment for a variety of enveloped viruses (219). The exposure of PS on the surface of the PM of apoptotic cells (220) is important for their clearance by phagocytes, and it is thought that diverse enveloped viruses hijack PS exposure to facilitate attachment and entry into target cells (219). TIM family members are variably expressed on myeloid and activated T cell subsets. In the case of HIV-1, expression of TIM-1 in target cells enhances virion entry. This may be by upregulating CD4/coreceptor levels, but very recent evidence has shown that PS exposure on the target cell is important for HIV-1 fusion. Conversely, overexpression of TIM family members restricts virion release by mediating a phenotype remarkably similar to tetherin (220). Of note, TIM-3 silencing in primary macrophages enhances virion release 2–4 fold, suggesting these observations maybe of relevance *in vivo*. Interestingly, the mucin domain of TIM-1 is highly polymorphic and homozygosity for a 6 amino acid in-frame deletion variant (delMTTTVP) has been associated with reduced HIV-1 disease progression (221) and replication in *ex vivo* cultured CD4+ T cells (222). Whether this is because of an inhibitory effect or a reduced entry-enhancing activity is yet to be determined.

The inhibition of processing and incorporation of Env into nascent virions was suggested as an antiviral mechanism of IFN-I against HIV-1 many years ago (223). Recent studies have implicated this process as a target for two ISGs (224, 225). LGALS3BP/90K, a cysteine rich secreted scavenger receptor that has a role in regulating cell adhesion, is strongly upregulated by IFN-I and IFN-II and is present at high concentrations in most bodily fluids. Expression of cell-associated 90K blocked envelope incorporation and gp160 processing dependent on its BR-C, ttk, BOZ/Poxvirus Zinc finger (BTB/POZ) domain (225). 90K does not generally inhibit furin-like proteases that cleave a number of viral glycoproteins, nor does it have antiviral activity against murine retroviruses. Neither was 90K found to directly associate with gp160 in the secretory pathway. However, 90K depletion in both T cells and macrophages enhanced HIV-1 replication. A similar activity has been associated with guanylate binding protein 5 (GBP5), a member of a family of IFN-induced GTPases (224). As with 90K, expression of GBP5 blocked the processing and incorporation of gp160 as well as other retroviral envelope proteins. This required the ability of GBP-5 to localize to the Golgi network, but appears independent of its GTPase activity. Furthermore, GBP5 expression levels in primary macrophages inversely correlated with viral replication. Interestingly, Env expression levels were a key to HIV-1 GBP-5 sensitivity. Mutations in the start codon of *vpu*, which is expressed from the same mRNA, enhances Env expression levels and confers partial GBP5 resistance. Since Vpu is essential to counteract tetherin (see below), the authors speculate that balancing the expression of Vpu and Env allows for optimal viral replication in the face of these two IFN-induced restrictions. As yet, little further mechanistic understanding of 90K or GBP-5-mediated effects on Env are known, or indeed whether they are related given their phenotypic similarities.

The assembly and budding of the nascent virion at the PM has been suggested as a target for IFN-I-mediated restriction. 2′,3′-cyclic-nucleotide 3′-phosphodiesterase (CNP) was identified in an overexpression screen of ISGs that restrict viral release (226). CNP, a membrane-associated enzyme, bound to Gag in membrane fractions and inhibited particle formation independent of its enzymatic activity. While most mammalian CNP orthologues tested had antiviral activity against HIV-1, a single amino acid difference in murine CNP accounted for its lack of retroviral restriction. Selection of CNP-resistant viruses resulted in a single point mutation (E40K) in the matrix (MA) domain of the Gag polyprotein, which alongside the murine CNP species-specific difference, governed CNP/Gag interactions. Interestingly, the equivalent position in MA is a K in some HIV-2 and SIV isolates and this correlates with their resistance to CNP. However, whether CNP ever gets the opportunity to restrict HIV-1 *in vivo* is unclear. It is expressed mainly in oligodendrocytes and epithelial cells, with some expression in DCs, but is not detectable in primary CD4+ T cells.

Finally, the ESCRT-mediated release of the virus has been suggested as a target of IFN-mediated restriction. The interferon-induced ubiquitin-like modifier, ISG15, has a broad role in antiviral defence (227). The ESCRT-III complex constricts the neck of the budding virion to the point of scission. This requires the polymerization of its charged multivesicular protein (CHMP) components into helical polymers on the internal surface of the neck, followed by their regulated disassembly by the AAA-ATPase VPS4 and its cofactor, LIP5 (4). Direct conjugation of ISG15 (ISGylation) to various CHMPs blocks their interaction with VPS4/LIP5, thereby stalling retrovirus budding (228, 229). ISGylation of CHMP5 appears to be essential for this process as in its absence, no other CHMP becomes modified (228). CHMP5 is dispensable for ESCRT-III function itself, raising the possibility that it is a regulator that can rapidly inhibit ESCRT function after IFN treatment. Whether CHMP5 ISGylation is a major mechanism of antiretroviral defence under physiological conditions is not yet clear. Another ESCRT-III regulating factor, CC2D1A, binds to the ESCRT-III CHMP4B and blocks polymer formation, thereby dominantly interfering with HIV-1 assembly (230, 231). CC2D1A itself is an ISG (7), although whether it acts in a directly antiviral capacity is not known given that it has also been identified as a regulator of TBK1, a major kinase in the pattern recognition signaling cascade (232).

## Concluding Remarks

Negotiating the limiting membranes of the cell represent the first and last stages of HIV-1 replication. As analogous processes are common to all enveloped viruses, the evolution of antiviral factors that inhibit them present general first line defences against HIV-1 and related viruses. Their importance is reflected in the resistance mechanisms that primate lentiviruses have evolved to avoid them, and the evidence that their antiviral activities present significant barriers to viral transmission, systemic spread and augmentation of other immune responses. This suggests that targeting the virus’s resistance to PM-based host restrictions may have therapeutic or vaccine-relevant potential. Their study also reveals fundamental new understanding of the basic processes of viral entry and exit from the cell.

## Author Contributions

All authors listed have made a substantial, direct, and intellectual contribution to the work and approved it for publication.

## Conflict of Interest Statement

The authors declare that the research was conducted in the absence of any commercial or financial relationships that could be construed as a potential conflict of interest.
